# Consensus recommendations for diagnosis, management and treatment of Fabry disease in paediatric patients

**DOI:** 10.1111/cge.13546

**Published:** 2019-06-06

**Authors:** Dominique P. Germain, Alain Fouilhoux, Stéphane Decramer, Marine Tardieu, Pascal Pillet, Marc Fila, Serge Rivera, Georges Deschênes, Didier Lacombe

**Affiliations:** ^1^ Division of Medical Genetics University of Versailles Montigny France; ^2^ Metabolic Diseases Unit HFME University Hospital Lyon Lyon France; ^3^ Paediatric Department, Inserm U1048 Toulouse University Hospital Toulouse France; ^4^ Paediatric Department Tours University Hospital Toulouse France; ^5^ Paediatric Department Bordeaux University Hospital Pellegrin Bordeaux France; ^6^ Department of Paediatric Nephrology—Montpellier University Arnaud de Villeneuve Hospital Montpellier France; ^7^ Department of Paediatric Neurology Bayonne Hospital Bayonne France; ^8^ Department of Paediatric Nephrology Paris University Hospital Robert Debré Paris France; ^9^ Department of Medical Genetics, CHU Bordeaux INSERM U1211 Université de Bordeaux Bordeaux France

**Keywords:** children, diagnosis, enzyme replacement therapy, Fabry disease, management, paediatric, treatment

## Abstract

Fabry disease (FD), a rare X‐linked disease, can be treated with bi‐monthly infusion of enzyme replacement therapy (ERT) to replace deficient α‐galactosidase A (AGAL‐A). ERT reduces symptoms, improves quality of life (QoL), and improves clinical signs and biochemical markers. ERT initiation in childhood could slow or stop progressive organ damage. Preventative treatment of FD from childhood is thought to avoid organ damage in later life, prompting a French expert working group to collaborate and produce recommendations for treating and monitoring children with FD. Organ involvement should be assessed by age 5 for asymptomatic boys (age 12‐15 for asymptomatic girls), and immediately for children diagnosed via symptoms. The renal, cardiac, nervous and gastrointestinal systems should be assessed, as well as bone, skin, eyes, hearing, and QoL. The plasma biomarker globotriaosylsphingosine is also useful. ERT should be considered for symptomatic boys and girls with neuropathic pain, pathological albuminuria (≥3 mg/mmol creatinine), severe GI involvement and abdominal pain or cardiac involvement. ERT should be considered for asymptomatic boys from the age of 7. Organ involvement should be treated as needed. Early diagnosis and management of FD represents a promising strategy to reduce organ damage, morbidity and premature mortality in adulthood.

## Graphical abstract

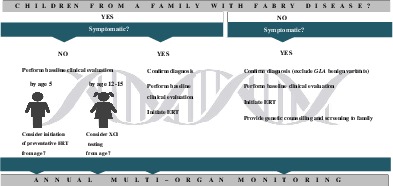

## 1 INTRODUCTION

Fabry disease (FD; OMIM#301500) is a rare X‐linked lysosomal disease caused by pathogenic variants in the *GLA* gene. The resulting deficiency of the lysosomal enzyme α‐galactosidase A (AGAL‐A) leads to accumulation of globotriaosylceramide (Gb_3_) and its derivative sphingoid base, globotriaosylsphingosine (LysoGb_3_), in the lysosomes of virtually all cell types of the body.^1^ FD is a multisystem disease, with the most serious clinical impact observed in the heart, kidneys and central nervous system (CNS).^2^, ^3^

FD was traditionally considered to be an adult disease, but it is now recognised that disease processes and symptoms start in infancy or early childhood. Early manifestations of classic FD in children include pain (dysesthesia), reduced or absent sweating (hypohidrosis or anhidrosis), corneal whorls (*cornea verticillata*), angiokeratoma and gastrointestinal (GI) discomfort.^4^ Pain represents the most striking symptom in children with FD, but overall quality of life (QoL) is often considerably impacted and characterised by fatigue, anxiety, depression, and school absences.^5^ Later, adults can experience progressive renal disease, and cardiac and central nervous system (CNS) complications which contribute to morbidity and early mortality.^6^

FD can manifest with a range of severities and has a spectrum of phenotypes. In classic FD, the first symptoms, including chronic neuropathic pain and episodic severe pain crises, typically emerge during childhood. Symptoms such as fatigue, hypohidrosis, skin angiokeratomas, diarrhoea, abdominal pain, hypoacousia, tinnitus, and *cornea verticillata* are additional common early manifestations. Occult kidney injury may occur at a young age, including pathological albuminuria and glomerulosclerosis. Symptomatic organ complications typically emerge in young adult patients, including chronic kidney disease (CKD) progression to renal failure and left ventricular hypertrophy (LVH) associated with myocardial fibrosis and arrhythmias,^7^ pulmonary involvement,^8^ sudden deafness^9^ transient ischemic attacks, strokes, and eventually premature death. Non‐classic phenotypes of FD include later‐onset forms with predominant cardiac involvement due to pathogenic *GLA* variants such as p.Phe113Leu or p.Asn215Ser, the most frequent ones in Caucasian subjects, or IVS4+919G>A, highly prevalent in Chinese‐Taiwanese populations. Later‐onset phenotypes are frequently under‐recognised because they lack classic manifestations of FD, such as acroparesthesia, *cornea verticillata* or angiokeratoma.^6^ Screening studies of high risk populations (eg, patients with left ventricular hypertrophy or on haemodialysis) have identified previously undiagnosed FD in adults aged from 30 years up.^10^, ^11^, ^12^, ^13^ Routine screening of these at‐risk populations may identify new cases, allowing initiation of effective treatment.

The natural history and characteristics of FD in children has been clarified in recent years by studies of patient registries. Symptoms appear at a median age of 6 years in boys and 7‐8 years in girls.^14^, ^15^ The age of symptom onset was higher in a survey of index cases with no known family history of FD (10.9 years in boys and 22.6 years in girls).^16^ The non‐specific nature of FD symptoms in children can significantly delay diagnosis in index patients. Indeed, the delay between symptom onset and diagnosis of FD has been reported as 13.7 years in males and 16.3 years in females.^16^

The longer time to diagnosis in females arises in part from disease heterogeneity associated with an X‐linked disease but also from the now‐discredited assumption that females with *GLA* pathogenic variants were merely carriers of FD.^17^ It is now known that females can occasionally have severe FD, similar to the classic FD phenotype, seen most commonly in hemizygous boys with dramatically decreased (<1%) or no AGAL‐A activity.^18^ In classic FD, the phenotype and natural disease course in females is mainly determined by the pattern of X‐chromosome inactivation (XCI).^19^ Severe classic phenotype of FD arises in females when XCI pattern is skewed towards the mutant *GLA* allele in a ratio of 80:20 or greater across tissues. Echevarria et al found skewed XCI in 16 of 55 (29%) adult female FD patients. Of these patients, 10 had XCI skewed towards the mutated *GLA* allele, with low or absent residual enzyme activity and higher clinical severity that increased with age.^19^

### 1.1 Treatments

In 2001 two forms of enzyme replacement therapy (ERT) were approved by the European Medicines Agency (EMA). Both forms of ERT are lifelong treatments and are administered by intravenous infusion every other week. Agalsidase alfa (Replagal, Shire, Cambridge, Massachusetts) is approved for children and adolescents aged 7 years and older at a dose of 0.2 mg/kg.^20^ Agalsidase beta (Fabrazyme, Sanofi‐Genzyme) is approved for children and adolescents aged 8 years and older at a dose of 1.0 mg/kg.^21^ To date, agalsidase beta is the only FDA‐approved ERT for FD.

A recent Cochrane review of evidence from randomised controlled trials shows that, compared to placebo, ERT reduces plasma Gb_3_ and improves pain‐related QoL.^22^ The Cochrane review was complemented by a pooled analysis of cohort studies, and showed that patients taking agalsidase beta had a significantly lower incidence of cardiovascular, renal and cerebrovascular events than patients not taking ERT.^23^

Various open‐label studies have showed the efficacy and safety of ERT in children: ERT treatment decreases Gb_3_ accumulation in tissue, plasma and urine,^24^, ^25^, ^26^ and improves pain^27^ and GI symptoms,^24^, ^28^, ^29^ as well as QoL, energy and activity levels.^26^, ^29^, ^30^

A long‐term study of ERT efficacy in adults reported a 10‐year survival rate of 94% and most patients (81%) had no severe clinical events during this time. Younger patients (mean age at agalsidase beta onset: 25 years) demonstrated the most benefit from ERT, and renal decline was slower in those patients, suggesting that early initiation of ERT could slow or even prevent renal decline.^31^

An additional drug, the pharmacological chaperone migalastat, was approved in the EU in 2016 and in the USA in 2018. Migalastat is a small‐molecule inhibitor of AGAL‐A which, when bound at sub‐inhibitory concentrations, corrects the folding error of some missense *GLA* variants thereby facilitating normal trafficking of AGAL‐A from the endoplasmic reticulum through the Golgi to the lysosome.^32^ Once in the lysosome, the complex dissociates, leaving functional AGAL‐A to degrade accumulated substrate.^33^ In the phase III FACETS trial (NCT00925301), migalastat did not achieve its primary endpoint vs placebo. After 24 months of treatment, in a post hoc analysis of the modified intent to treat (ITT) population, migalastat was associated with stabilisation of kidney function and improvement of left ventricular mass index^34^ in adult patients with FD and amenable *GLA* variants.^34^ Migalastat, which indication is limited to adult Fabry patients with amenable *GLA* variants, is not currently approved in children under the age of 16 in the absence of paediatric data.

### 1.2 Existing guidelines

Although several guidelines and consensus recommendations exist for FD^35^, ^36^, ^37^, ^38^ there has been a shift in mindset over the past decade. This was prompted by better understanding that early ERT initiation can prevent organ damage in later life, improving both morbidity and mortality. The philosophy has changed from treatment to prevention, with the aim of maintaining organ function, optimising QoL and preserving life expectancy. There is consequently a need for updated recommendations for managing children with FD.

A French working group, comprising paediatricians and geneticists experienced in treating FD, convened three times between 2016 and 2017 to discuss the previous French guidelines^36^ in light of developments in the field, and to discuss a management strategy for children with FD.

Here we present the proposals agreed by the group as a practical guide for diagnosis, treatment and monitoring of children with FD.

## 2 DIAGNOSIS IN CHILDREN

FD is generally thought to be underdiagnosed. Recent screening studies report that prevalence of FD in males may be as high as 1:3100^39^ and 1:8500.^40^ These results are significantly higher than previous estimates of 1:117 000^41^ and 1:476 000.^42^ The estimate of 1:3100^38^ included five newborns diagnosed with *GLA* variants which are not disease‐causing (p.E66G, p.A143T—three cases, p.R118C). Exclusion of these patients reduces the incidence to an estimate of 1:5000 (DP Germain, unpublished data). Population‐wide newborn screening is not currently implemented in any European country except Italy, while Taiwan and several states in the United States systematically screen newborn children for several lysosomal disorders including FD.^38^, ^43^

### 2.1 Children from families with known Fabry disease

Systematic screening of children with at least one first degree relative with FD is the simplest and fastest way to improve the rate of diagnosis. In France, FD in such children is usually detected by a geneticist or a paediatrician. In boys, the diagnosis is confirmed by nearly complete deficiency of AGAL‐A activity in leukocytes, dried blood spot or plasma^36^ along with confirmation of a pathogenic *GLA* variant. In girls, however, the diagnosis is more complex because XCI in heterozygotes can result in only partially deficient or even subnormal AGAL‐A levels in the leukocytes and plasma.^19^ Thus, the diagnosis of FD should always be confirmed by identification of a pathogenic *GLA* variant.^36^, ^44^

Of note, predictive genetic testing of children born to parents with FD is considered to be justified, according to the European Society of Human Genetics guidelines, because FD is now recognised to have onset in childhood and ERT could prevent damage in adulthood.^45^

Prenatal diagnosis of FD is available in France for male, but generally not for female, foetuses. This discrepancy is for ethical reasons related to the heterogeneity of disease severity in females. Moreover, families with known FD can be offered pre‐implantation genetic diagnosis (PGD) of embryos prior to implantation during assisted reproduction, but this remains rare.

### 2.2 Diagnosis of index cases

Owing to the lack of specific symptoms, diagnosis of index cases of FD is usually delayed and rarely occurs during childhood. The main signs and symptoms of FD in childhood are pain, fatigue, GI problems, reduced or absent sweating, heat/cold and exercise intolerance and angiokeratoma.^14^ Other symptoms include the presence of corneal whorls upon slit lamp examination,^46^ hearing loss,^47^ tinnitus,^48^ pathological albuminuria, delayed growth and reduced QoL. The signs and symptoms that should alert paediatricians to the possibility of FD are presented in Table 1, along with their frequency and age of onset.

**Table 1:** Signs and symptoms of FD in children according to the literature

Sign/symptom	Frequency	Age of onset
**Pain** Dysesthesia/episodic crisis of burning in hands or feet	49.7% (58.8% boys, 40.5% girls)^14^ 72.3%^47^ 66% (67% boys, 65% girls)^4^ 63% (67% boys, 59% girls)^15^	2‐4 years^52^ Median: 7 years boys, 9 years girls^14^ 10.1 years boys,^3^ 15 years girls^2^ Median: 7.2 years boys, 8.3 years girls^15^
**Reduced sweating** Hypohidrosis or anhidrosis (not sweating enough)	25.3% (28.4% boys, 22.2% girls)^14^ 59% (93% boys, 25% girls)^4^ 43.1% (69% boys, 17.2% girls)^15^	2.5 years^52^ Hypohidrosis (median) 10.1 years boys, 4.2 years girls^15^ Anhidrosis (median): 7.8 years boys, no cases for girls
**Corneal signs** Corneal whorls/cornea verticillata	71.5% (73% boys, 70% girls)^4^ 50%^46^ Cornea verticillata: 50.8% (36.1% boys, 65.5% girls)^14^	Newborn^52^ Median (range): 8.1 (2‐15) years^46^ Cornea verticillata (median) 12.1 years boys, 8.9 years girls^15^
**Gastrointestinal symptoms** Nausea, vomiting, non‐specific abdominal pain, constipation, diarrhoea	17.9% (23.2% boys, 11.4% girls)^14^ 30% (boys 40%, girls 20%)^4^ 49.8%^28^	Median: 5 years boys, 9.5 years girls^14^ 1‐4.1 years^52^ 1‐17.8 years^15^
**Heat/cold/exercise intolerance**	Heat: 25.3%, cold 14%^14^ Heat: 38.4% (38.9% boys, 37.9% girls)^15^ Cold: 17% (16.7% boys, 17.2% girls)^15^	3.5 years^52^ Heat (median) 7.4 years boys, 15.7 years girls^15^ Cold (median) 5.0 years boys, 7.7 years girls^15^
**Hearing problems** Hearing loss, tinnitus and vertigo	Confirmed hearing loss: 19%,^48^ 12.8%,^47^ 21.8% (19.4% boys, 24.1% girls)^15^ Tinnitus: 31.8%,^48^ 40% (22.2% boys, 41.3% girls)^15^ Vertigo 25.5%,^48^ 30.4% (19.4% boys, 41.4% girls)^15^	4 years^52^ Tinnitus (median): 10 years boys, 13.9 years girls^15^ Vertigo (median): 11.8 years boys, 13.4 years girls^15^ Hearing loss (median): 2.7 years boys, 14.4 years girls^15^
**Angiokeratomas**	14.2% (19.6% boys, 7.6% girls)^14^ 43.5% (boys 57%, girls 30%)^4^ 40% (42% boys, 38% girls)^15^	Median: 7 years boys, 9.5 years girls^14^ Median: 9.1 years boys, 14.4 years girls^15^
**Renal signs** Hyperfiltration, pathological albuminuria, proteinuria	Hyperfiltration: unknown Microalbuminuria: 13.2%^15^ Proteinuria: 19.7%^15^	Microalbuminuria (median): 16.5 years boys, 15.9 years girls^15^ Proteinuria (median): 13.8 years boys, 14.1 years girls^15^
**Cardiac signs** Conduction abnormalities, valvular dysfunction, arrhythmias	Conduction abnormalities: 7.6% (8.3% boys, 6.9% girls),^15^ 6.8% (9.7% boys, 3.8% girls)^14^ Valve disease: 14.9% (5.6% boys, 24.1% girls),^15^ 18.3% (22.6% boys, 13.9% girls)^14^ Arrhythmias: 1.4% (2.8% boys, 0% girls),^15^ 4.9% (7.3% boys, 2.5% girls)^14^	Conduction abnormalities: (median): 10.3 years boys, 16.9 years girls^15^ Valve disease: (median): 8.6 years boys, 14.4 years girls^15^ Arrhythmias: (median): 9.3 years boys^15^

Abbreviation: FD, Fabry disease.

FD should also be considered (using cascade genetic testing) if any family members, especially males, died before age 50 due to stroke, cardiac arrhythmias, cardiac conductance problems or renal insufficiency.^36^

A hallmark sign of classic FD in children is neuropathic pain in the hands and feet, most commonly in the palms, soles and fingertips.^49^ Neuropathic pain has been reported by up to 72.3% of patients with FD^4^, ^14^, ^47^ and is more frequently present in boys. The pain is most commonly described as “burning” followed by stabbing, tingling and shooting and triggered by physical exercise, heat and fever.^49^ Patients can experience crises of excruciating pain that radiate proximally to the entire body and that can last up to several days, with no respite offered by analgesics.^49^, ^50^ The mean age of onset of pain has been reported as 10.1 years in boys^3^ and 15 years in heterozygote girls^2^ although one study reported median onset of neuropathic pain as 7 years in boys and 9 years in girls.^14^

However, pain in children can also arise from diverse conditions such as growing pains. It can therefore be difficult for physicians to correctly attribute pain to a diagnosis of FD, especially in the absence of other symptoms or prior family history of FD that could alert physicians to the possibility of FD. Growing pains can be further differentiated from FD pain by assessing when and where the pain occurs: FD pain is most frequently experienced as burning, stabbing, tingling or shooting pain in the hands and feet and occurs during the day, particularly in late morning. In contrast, growing pains usually occur in the lower extremities during the late evening and night.^51^ In addition to neuropathic pain, children with FD often experience GI symptoms such as abdominal pain and diarrhoea.^52^ These symptoms are seen early in childhood with a median onset of 5 years in boys and 9.5 years in girls and constitute the initial symptoms of classic FD in up to 19% of patients.^13^

Misdiagnosis of index cases is common with up to 25% of patients experiencing a previous misdiagnosis^16^ such as rheumatological disease, arthritis, dermatomyositis, erythromelalgia, Osler's disease, neuropsychological disease, Ménière's disease, irritable bowel syndrome (IBS), Raynaud's syndrome, growing pains,^16^ acute appendicitis, lupus, or multiple sclerosis.^53^ This is often a challenging and chaotic time for families, with many hospital visits with different specialists.

### 2.3 Announcing the diagnosis

For index cases, the diagnosis should be announced to the family according to Good Practice guidelines.^54^ The disease is explained, and the family is supported for the next steps, including genetic counselling.^55^ A detailed genealogy/family history is performed to screen/identify other cases in the same family.^55^

## 3 BASELINE CLINICAL WORK‐UP AND SUBSEQUENT MONITORING

Following diagnosis of FD, a baseline assessment of organ involvement should be performed, followed by annual monitoring in boys and every 2‐3 years in girls. A multi‐disciplinary approach with specialists experienced in the care of FD patients is essential. The multi‐disciplinary team should include the following specialists: paediatrician, geneticist, pain specialist with intervention from other specialists as required.^36^ Advice can be obtained from the national tertiary referral centre (http://www.centre-geneo.com).

**Figure 1:**
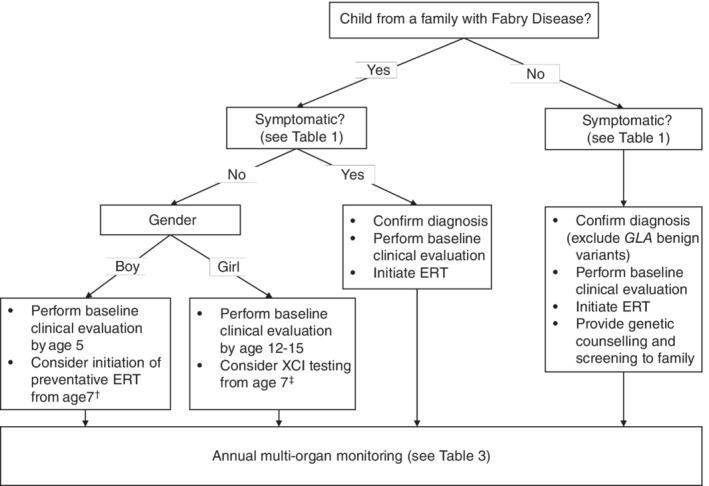
Summary of recommendations for diagnosis and monitoring of Fabry Disease. †Early initiation of preventative ERT can be considered for asymptomatic boys based on the criteria defined earlier in this paper. ‡Girls aged 7 and over should be offered XCI testing and in case of skewing in favour of expression of the mutant GLA allele be considered for ERT. Abbreviations: ERT, enzyme replacement therapy; XCI, X chromosome inactivation

For asymptomatic children from families with FD, we recommend that the baseline assessment of organ involvement occurs no later than 5 years of age for boys and 12‐15 years of age for girls (Figure 1). Parents of asymptomatic FD children should also be educated to recognise the major signs and symptoms of FD in young children (Table 1) and to seek medical advice if symptoms manifest. For symptomatic children, whether from families with FD or newly diagnosed index cases, the baseline assessments should be performed immediately after diagnosis.

The procedures to be performed at baseline and subsequently at annual monitoring in boys or every 2 to 3 years in girls are presented in Table 2. In the case of later‐onset phenotypes, the monitoring procedures should be focused on the affected organ (ie, heart) and its periodicity should be determined case by case.^56^ In children diagnosed with pathogenic *GLA* variants responsible for later‐onset phenotype, through either screening protocols or cascade genetic testing within families, particular attention should be given to appropriate genetic counselling and the delivery of detailed information on the natural course of this form. Because later‐onset FD often become overt only in adulthood, patient monitoring should be adapted and unnecessary procedures and treatment avoided.

**Table 2:** Procedures to be carried out at initial clinical workup and follow‐up monitoring

System	Assessment	Baseline assessments†	Monitoring (annual in boys, every 2‐3 years in girls, unless otherwise indicated)
Kidneys	Serum urea	X	X
	Serum creatinine	X	X
	Serum uric acid	X (for children aged >5 years)	X (for children aged >5 years)
	Estimated GFR (composite Schwartz formula 2012)	X (for boys and girls)	X (if no renal involvement at baseline)
	Cystatin C	X	X
	Measured GFR (if available)	X (for boys)	X (for boys and girls with renal involvement at baseline)
	Albuminuria	X	X
	Proteinuria	X	X
	Kidney ultrasound	X	—
Heart	ECG	X	X
	Cardiac ultrasound	X	X
	Holter	—	If indicated by baseline ECG; optional depending on symptoms and clinical availability of test from aged 15
	Cardiac MRI with T1 mapping	Consider if technique is available and sedation of patient not necessary	
Nervous system	Cerebral MRI	No (consider at adulthood)	
	Pain consultation	If pain is present and if needed	
Metabolism	α‐galactosidase A activity in leucocytes, plasma or DBS	At diagnosis	—
	Plasma lysoGb_3_	Before ERT initiation	X
	Anti‐agalsidase antibodies	Before ERT initiation	X
Eyes	Ophthalmologic exam including slit lamp examination	X	—
Skin	Clinical examination	X	X
	Standardised photos of angiokeratomas	X	X
Gastrointestinal/endocrinology	Clinical examination and growth curve	X	X
Other	25OHD3	X	X
	QoL and school absenteeism	X	X
	Audiogram	X (from age 10)	If indicated by symptoms
	X chromosome inactivation	X (for girls)	—

Abbreviations: DBS, dried blood spot testing; ECG, electrocardiogram; ERT, enzyme replacement therapy, lysoGb_3_, globotriaosylsphingosine; GFR, glomerular filtration rate; MRI, magnetic resonance imaging; 25OHD3, 25‐hydroxyvitamin D3.

† The baseline audit of organ involvement should be performed following diagnosis for symptomatic children by age 5 in asymptomatic boys and by age 12‐15 for asymptomatic girls.

### 3.1 Renal assessment

In general, children do not suffer from renal insufficiency until adulthood, when renal failure accounts for much of the morbidity and mortality associated with FD, particularly in males.^57^ Accumulation of Gb_3_ in kidney cells and podocytes foot process effacement can be observed in kidney biopsies from children with FD, even before pathological albuminuria and proteinuria manifest as early signs of renal involvement.^25^, ^58^, ^59^ Performing routine renal biopsy in children remains an area of debate within the community of physicians involved in the care of patients with FD.^57^ Renal biopsy has been proposed and shown to be safe by several authors^57^, ^58^, ^59^, ^60^ and should be considered in selected paediatric cases, particularly when the decision to start ERT is questioning or in children with significant proteinuria or decline in renal function; in whom renal biopsy is essential to rule out a second renal disease.^38^

We recommend that albuminuria, proteinuria and GFR be assessed in all cases. In clinical practice, GFR can be estimated using the composite Schwarz formula/equation^61^ rather than using the single classic markers of renal function. eGFR can be computed directly using height, serum creatinine, blood urea and serum cystatin C on https://www.kidney.org/professionals/KDOQI/gfr_calculatorPed.^62^ The size of the kidneys should also be measured by ultrasound at the baseline assessment.

### 3.2 Cardiac assessment

The major sign of cardiac involvement in FD is left ventricular hypertrophy (LVH), but conduction disturbance, such as short PR interval (due to accelerated conduction in the absence of accessory pathway), and rhythm disturbances, such us sinus bradycardia, have also been reported early in life. The benefit of ERT on conduction defects is currently unknown.^6^ Of note, new reference centiles have recently been published for calculating left ventricular mass in children with low body weight, as is often the case with children with FD.^63^ Cardiac ultrasound and ECG are indicated at the baseline assessment and at all monitoring visits. The Holter test is recommended only if indicated by symptoms, because severe arrhythmias are not usually seen in early childhood. Magnetic resonance imaging (MRI) T1 mapping can also be considered if indicated by symptoms and in children old enough to undergo the test without sedation^62^

### 3.3 Nervous system assessment

Brain MRI to screen for white matter lesions is not indicated in children, except if the patient exhibits clinical signs suggestive of neurological impairment. Consultation with a pain specialist can be considered if needed at the baseline assessment or any monitoring visits.

### 3.4 Ophthalmological assessment

A complete ophthalmological assessment is recommended at the baseline assessment. The presence of *cornea verticillata* (vortex keratopathy or whorls) strongly supports the diagnosis of a classic phenotype of FD and is widely considered as a hallmark of classic FD.^64^

### 3.5 Dermatological assessment

A dermatological assessment of angiokeratoma is recommended at the baseline visit and at subsequent monitoring assessments in case of worsening of symptoms, along with standardised photographs of angiokeratomas if possible.

### 3.6 Gastrointestinal assessment

At baseline and all monitoring visits, a GI assessment is recommended, along with establishment of a growth curve (using height and weight).

### 3.7 Metabolic assessment

Alpha‐galactosidase A activity should be assayed at baseline. Measurement of urinary Gb_3_ had being historically proposed^65^ and had shown an ERT dose‐dependence response,^66^ but its clinical relevance has not been elucidated. Consequently, plasma lysoGb_3_ may prove a better biomarker of disease activity and its monitoring is currently preferred and recommended.^30^, ^67^, ^68^, ^69^. If ERT is initiated, anti‐agalsidase antibodies should be regularly monitored.^70^

### 3.8 Other assessments

QoL assessment using the paediatric Quality of Life Inventory (peds QL)^5^ and an assessment of school life (including absenteeism) should be performed at the baseline assessments and at all monitoring visits.

An audiogram should be performed at age 10 (or at the baseline assessment if diagnosis is after age 10). Annual audiograms are not recommended but can be performed if symptoms indicate it to be necessary.

To mitigate the increased risk of osteoporosis in adults with FD^71^ and aligned with the experimental evidence of nephroprotective action of vitamin D receptor activation,^72^ children should also be monitored for vitamin D deficiency by measurement of serum 25OHD3.

XCI should be assessed in girls at the baseline visit.^19^

## 4 TREATMENT

### 4.1 ERT

In France, ERT initiation for Fabry disease must be approved by a tertiary referral centre. Initiation of ERT in childhood could slow, or even, stop the progression of organ damage before irreversible changes occur.^22^, ^23^ ERT should be considered for symptomatic boys and girls with neuropathic pain, pathological albuminuria (at least 3 mg/mmol creatinine), severe GI involvement and abdominal pain or cardiac involvement. There are currently no data supporting ERT initiation based on the sole presence of angiokeratoma. For asymptomatic boys, ERT initiation criteria currently follows the current Summary of Product Characteristics (SmPC) that indicates treatment initiation from the age of 7 or 8 years or over, in the absence of data in younger population. However, it is the opinion of this group that asymptomatic boys may benefit from an earlier initiation of ERT based on the following criteria: presence of a pathogenic *GLA* variant responsible for the classic phenotype, family history of disease severity in males, undetectable AGAL‐A activity in peripheral blood leukocytes and plasma lysoGb_3_ over 20 nmol/L. There is currently no data supporting ERT initiation in asymptomatic girls but in the opinion of the authors, girls heterozygotes for FD and aged 7 and over should be offered XCI testing and, in case of skewing in favour of expression of the mutant *GLA* allele, equally be considered for ERT (Table 3).

**Table 3:** Treatment of symptoms in children with Fabry disease

Target	Treatment in children
Underlying disease	ERT
Pain	ERT, anti‐epileptics, tricyclic antidepressants and serotonin‐norepinephrine uptake inhibitors
Pathological proteinuria	ERT, ACE inhibitors or ARBs
Hearing loss	Hearing aids, cochlear implant
Cardiovascular disease prevention	Healthy eating, avoidance or cessation of smoking, management of dyslipidaemia and blood pressure using statins if necessary
Gastrointestinal disturbances	ERT, dietary restrictions, small meals
Psychological problems	Psychological support

Abbreviations: ACE, angiotensin enzyme converting; ARBs, angiotensin receptor blockers; ERT, enzyme replacement therapy.

ERT should be dosed according to the SmPC. Treating physicians and parents should discuss the choice of infusion: either via a peripheral catheter or a central access port due to a lack of venous access. Patients should be monitored for infusion reactions, and anti‐agalsidase antibody titres should be performed before ERT initiation and every 6 months thereafter.^70^

Treatment initiation in later‐onset phenotype of FD should be adapted to the natural history of this form which often reveal only in adulthood. Detailed explanation should be provided in order to avoid generating anxiety in families.

Moreover, careful consideration should be given to confirm or establish the pathogenicity of the identified *GLA* variant.^73^ In particular, pLeu3Pro (DP Germain, unpublished data), p.Glu66Gln, p.Arg118Cys,^74^ p.Ser126Gly (DP Germain, unpublished data), p.Ala143Thr,^75^ and p.Asp313Tyr^76^ are benign or likely benign *GLA* variants which should not be considered causative of FD. Consequently, specific therapy for FD should not be initiated in individuals bearing those non‐pathogenic *GLA* variants.

Initiation of ERT in children is a joint decision between the paediatrician or geneticist, patient and family after careful consideration of the protective benefits offered by ERT initiation vs the challenges of a lifelong bi‐weekly infusion regimen. Home infusion, which can alleviate the burden of biweekly infusions in the right circumstances, can be considered for patients who tolerate well the infusions at hospital.^36^

### 4.2 Pain management

Effective pain management is an important first step in improving QoL, pending the validation and initiation of ERT. The pain management strategy should be tailored to each child, with education on how to reduce or avoid triggers such as heat or strenuous exercise. In adults, chronic neuropathic pain is treated with anti‐epileptics,^36^, ^50^ tricyclic antidepressants and serotonin‐norepinephrine uptake inhibitors. Acute pain during crises is treated with intravenous anaesthetics, central analgesics and topical agents.^50^ No randomised controlled trial has compared the efficacy of different agents or optimal dosing for the management of Fabry pain in children. Nonetheless, recent US paediatric guidelines suggest an algorithm for starting dose, up‐titration and maximum doses of pain medication by age group.^38^ Children with pain should be started on low‐dose monotherapy, and slowly up‐titrated to the maximum dose, according to pain levels. If monotherapy proves to be ineffective after several weeks, a different pain medication can be added. In general, long‐term use of pain agents should be avoided due to neurological side effects. The use of pain medication should not delay initiation of ERT in symptomatic patients (Table 3).

### 4.3 Treating organ involvement

Pathological proteinuria can be treated with angiotensin enzyme converting (ACE) inhibitors or angiotensin receptor blocker (ARB).^35^ However, albuminuria ≥3 mg/mmol can be considered as an early sign of renal involvement which would be an indication for ERT initiation.

Cardiac changes can be observed from childhood, with significant morbidity in later life. We recommend implementing healthy lifestyle measures to prevent cardiovascular disease in later life, including healthy eating and management of blood pressure and dyslipidaemia, using statins if indicated.

Similarly, dietary measures can be implemented to prevent vitamin and iron deficiencies. GI involvement is common in children with FD and includes nausea, vomiting, non‐specific abdominal pain, constipation, diarrhoea. ERT with agalsidase beta has been shown to improve GI symptoms.^29^ Eating small, frequent meals and dietary restrictions can also help.^35^

## 5 PSYCHOLOGICAL SUPPORT

Diagnosis of FD in children is a difficult time and causes emotional distress for the entire family, as well as the patient. This distress can be magnified in families with known FD who understand the impact and progression of the disease. For index cases, a diagnosis of FD can represent medicalisation of the child, and indeed the entire family. In addition, maternal guilt and anxiety associated with the X‐linked transmission of FD can result in over‐protective parenting, further reducing QoL of the patient.

For children, the psychological burden of coping with FD, the restricted capacity to participate fully in vigorous games and sport can lead to difficulties including anxiety and depression.^77^ These challenges can lead to school absenteeism and decreased academic performance. Therefore, the multidisciplinary monitoring of children with FD should include an annual assessment of QoL and school attendance and performance. Children in difficulty should be referred to a psychologist or social worker for support.

The chronic nature of FD and regular monitoring means that a strong relationship can be formed between the child, their family and the treating physician. Transition to adult care should thus be handled with appropriate sensitivity, with a transition consultation provided to explain the handover to adult care.

In France, three patient organisations support people with FD: Association des Patients de la Maladie de Fabry (APMF), Vaincre les Maladies Lysosomales (VML), and Association pour l'Information et la Recherche sur les Maladies Rénales Génétiques (AIRG).

## 6 DISCUSSION

The understanding of FD has evolved considerably because ERT became available in 2001, partly due to the increased understanding of the natural disease course in children and adults. It is now understood that FD morbidity starts in childhood and worsens with age, and that some girls can have severe disease due to skewed XCI. Numerous studies suggest that early initiation of ERT, even before symptoms appear, can protect organs from damage in later life.

In general, existing guidelines recommend ERT initiation for all patients at the time of symptom onset^35^, ^36^ or organ involvement.^37^, ^78^ However, opinion diverges regarding the age of ERT initiation in asymptomatic boys, with recommendations ranging from 8‐10 years in the 2016 US guidelines^38^ to 10‐13 years in the 2006 international guidelines^32^ to 16 years in the 2015 European guidelines.^34^ During the shortage of agalsidase beta, a European group of experts recommended prioritisation of children for treatment allocation. Opinion is similarly divided for asymptomatic girls. In this paper, we recommend that ERT initiation should be considered for symptomatic boys and girls and that prophylactic ERT treatment be considered for asymptomatic boys since the age of 7 years old.

Estimates of the prevalence of FD have dramatically increased by around 10‐fold, based on a recent Italian newborn screening study.^39^ However, awareness of FD amongst medical professionals remains low. The diagnosis rate of FD must improve, given that specific therapies may protect major organs from progressive damage. There is an urgent need for more robust evidence for pain management strategies in children, and for long‐term evidence of the protective effect of ERT when initiated in childhood. Solutions to alleviate the burden of bi‐weekly infusions are also needed, and the role of anti‐agalsidase antibodies needs to be further elucidated in various patients' population subsets.

## 7 CONCLUSIONS

FD is a multisystemic disease that starts during childhood and worsens throughout adulthood. Boys with classic FD usually manifest with early onset symptoms impacting their quality of life during childhood. Girls usually have milder disease with later onset, but some girls may develop severe FD, similar to the phenotype observed in hemizygous boys. Early diagnosis and management of FD represent a promising strategy to reduce organ damage, morbidity and premature mortality in adulthood.

This paper provides expert consensus recommendations for the diagnosis, monitoring and treatment of children with FD (Figure 1) and a basis for updating the guidelines to reflect recent developments in the field.

## CONFLICTS OF INTEREST

D.P.G. is a consultant for Amicus, Sanofi‐Genzyme and Shire. He has received speaker's honoraria from Amicus, Sanofi‐Genzyme and Shire. A.F. has received speaker's and consultation honoraria from Amicus Therapeutics, Biomarin and Sanofi‐Genzyme. S.D. has received speaker's honoraria from Sanofi‐Genzyme, Shire and Amicus. M.T. received travel grants and speaker's honoraria from Sanofi‐Genzyme. P.P. received consultant honoraria from Sanofi‐Genzyme. M.F. received travel grants from Genzyme, Shire and Alexion. S.R. reports no conflict of interest. G.D. has received travel grants and inscription fees from Sanofi‐Genzyme and Alexion Pharma France. D.L. is a consultant for Sanofi‐Genzyme and has received travel grants and speaker's honoraria from Amicus, Biomarin, Shire and Sanofi‐Genzyme.

## AUTHOR CONTRIBUTIONS

Each author participated in the three expert panel meetings, participated in establishing consensus recommendations, revised and approved the manuscript. The first author made substantial contribution to the development of this manuscript.
